# PhyloAln: A Convenient Reference-Based Tool to Align Sequences and High-Throughput Reads for Phylogeny and Evolution in the Omic Era

**DOI:** 10.1093/molbev/msae150

**Published:** 2024-07-23

**Authors:** Yu-Hao Huang, Yi-Fei Sun, Hao Li, Hao-Sen Li, Hong Pang

**Affiliations:** State Key Laboratory of Biocontrol, School of Ecology, Sun Yat-sen University, Shenzhen 518107, China; State Key Laboratory of Biocontrol, School of Ecology, Sun Yat-sen University, Shenzhen 518107, China; State Key Laboratory of Biocontrol, School of Ecology, Sun Yat-sen University, Shenzhen 518107, China; State Key Laboratory of Biocontrol, School of Ecology, Sun Yat-sen University, Shenzhen 518107, China; State Key Laboratory of Biocontrol, School of Ecology, Sun Yat-sen University, Shenzhen 518107, China

**Keywords:** multiple sequence alignment, reference-based, phylogenetics, molecular evolution, omic data

## Abstract

The current trend in phylogenetic and evolutionary analyses predominantly relies on omic data. However, prior to core analyses, traditional methods typically involve intricate and time-consuming procedures, including assembly from high-throughput reads, decontamination, gene prediction, homology search, orthology assignment, multiple sequence alignment, and matrix trimming. Such processes significantly impede the efficiency of research when dealing with extensive data sets. In this study, we develop PhyloAln, a convenient reference-based tool capable of directly aligning high-throughput reads or complete sequences with existing alignments as a reference for phylogenetic and evolutionary analyses. Through testing with simulated data sets of species spanning the tree of life, PhyloAln demonstrates consistently robust performance compared with other reference-based tools across different data types, sequencing technologies, coverages, and species, with percent completeness and identity at least 50 percentage points higher in the alignments. Additionally, we validate the efficacy of PhyloAln in removing a minimum of 90% foreign and 70% cross-contamination issues, which are prevalent in sequencing data but often overlooked by other tools. Moreover, we showcase the broad applicability of PhyloAln by generating alignments (completeness mostly larger than 80%, identity larger than 90%) and reconstructing robust phylogenies using real data sets of transcriptomes of ladybird beetles, plastid genes of peppers, or ultraconserved elements of turtles. With these advantages, PhyloAln is expected to facilitate phylogenetic and evolutionary analyses in the omic era. The tool is accessible at https://github.com/huangyh45/PhyloAln.

## Introduction

Phylogenetic analyses clarify relationships and evolutionary histories across various species (Misof et al. 2014; Zeng et al. 2014; Prum et al. 2015), strains (Lemieux et al. 2021), individuals (Prüfer et al. 2014; Sun et al. 2023), cells (Coorens et al. 2021), and genes (Li et al. 2022). Furthermore, phylogenetic trees play a vital role in downstream applications, including ancestral state reconstruction, testing evolutionary hypotheses, and conducting phylogenetic comparative and diversity analyses, and consequently, they are widely used in research of evolutionary biology, conservation biology, earth history, and ecology (Mitchell et al. 2015; Lu et al. 2018; Liang et al. 2023; Siqueira et al. 2023). With the development of high-throughput sequencing technology, extensive omic data sets have been generated, including genomes, transcriptomes, amplicons, and plastomes. These data sets contribute significantly to the establishment of robust phylogenies across the tree of life. Examples include the construction of phylogenetic relationships among major lineages in insects (Misof et al. 2014), the evolutionary history of living birds (Prum et al. 2015), the highly supported genomic phylogeny of living primates (Shao et al. 2023), the position of the eukaryotes within Asgard archaea (Eme et al. 2023), and the deep phylogeny of angiosperms (Zeng et al. 2014). In addition, multiple sequence alignments (MSAs) conforming to standards for phylogenetic analyses find utility in various evolutionary studies beyond tree reconstruction. These MSAs can be employed in analyses such as the detection of selection pressure and site substitutions (Wang et al. 2020; Peng et al. 2023; Shao et al. 2023).

However, phylogenetic analyses relying on omic data, such as phylogenomic and phylotranscriptomic analyses, consistently involve intricate and time-consuming preparatory steps to generate MSA matrices, including data assembly from raw reads, contaminant identification, gene prediction, orthology assignment, MSA, and site trimming (Kapli et al. 2020). In these analyses, protein data sets derived from assembled omic data often serve as the basis for phylogenetic inferences. These inferences primarily rely on grouping proteins through *de novo* orthology assignments, exemplified by methods such as OrthoMCL (Li et al. 2003) and OrthoFinder (Emms and Kelly 2019). Moreover, omic data provide support for phylogenetic analyses employing sequences beyond nuclear protein–coding genes. Examples include the utilization of ultraconserved elements (UCEs) (Faircloth et al. 2012), ribosomal genes, mitochondrial sequences, and plastid sequences (Patwardhan et al. 2014), which require the corresponding methods to prepare their MSAs.

To better and more efficiently harness omic data, a strategy has been developed for generating target sequences or MSAs for phylogeny based on reference MSAs. Well-established ortholog databases, such as OrthoDB (Kuznetsov et al. 2023) and OMA (Altenhoff et al. 2024), offer extensive references for the tools with this reference-based strategy, such as Orthograph (Petersen et al. 2017) and Read2Tree (Dylus et al. 2024). Orthograph, primarily designed for phylotranscriptomics, employs a best reciprocal hit strategy to search transcriptomic sequences based on the globally best-matching cluster of orthologous genes (Petersen et al. 2017). However, Orthograph is limited to assembled sequences, and its output includes only target sequences without MSAs. In contrast, Read2Tree is a novel tool utilizing an assembly-free strategy that directly maps raw sequencing reads into groups of corresponding genes, generating consensus sequences, MSAs, and phylogenetic trees (Dylus et al. 2024). Nevertheless, Read2Tree is tailored for raw reads and features relatively inflexible processes, allowing only unaligned reference sequences and FASTQ read files as input. Additionally, these tools do not account for common foreign and cross-contamination, such as those from parasites, endosymbionts, or other species in the same sequencing batch, which can potentially impact phylogenetic analyses (Lusk 2014; Borner and Burmester 2017; Simion et al. 2018). Moreover, these tools may not adequately handle sequences distinct from nuclear protein–coding genes, including protein-coding sequences with nonstandard genetic codes (e.g. mitochondrial and plastid genes), non-protein–coding sequences (e.g. UCEs and ribosomal genes), and translated amino acid sequences.

In an effort to better address the requirements of phylogenetic and evolutionary analyses, this article introduces PhyloAln—a reference-based MSA tool designed for phylogeny and evolution. PhyloAln has the capability to directly map not only raw reads but also assembled or translated sequences onto reference MSAs. This feature makes it applicable to various omic data types, eliminating the need for the complex preparation involved in traditional methods, such as data assembly, gene prediction, orthology assignment, and MSA. Furthermore, PhyloAln is equipped to identify and eliminate foreign and cross-contamination within the generated MSAs—an aspect often overlooked in other reference-based methods. This capability enhances the quality of MSAs for subsequent analyses. To evaluate its performance, we subjected PhyloAln to testing with simulated data sets, including MSAs of single-copy orthologous genes spanning the tree of life and contaminated transcriptomes of fruit flies. Real data sets, consisting of MSAs from transcriptomes of ladybird beetles, plastid genomes of peppers, and UCEs of turtles, were also used for assessment. Comparative analyses with results from Read2Tree and Orthograph were conducted to assess the performance of PhyloAln. The assessments show that PhyloAln exhibits a relatively high accuracy in aligned sites and downstream phylogenetic analyses and broad availability in different data.

## Results

**Fig. 1.**
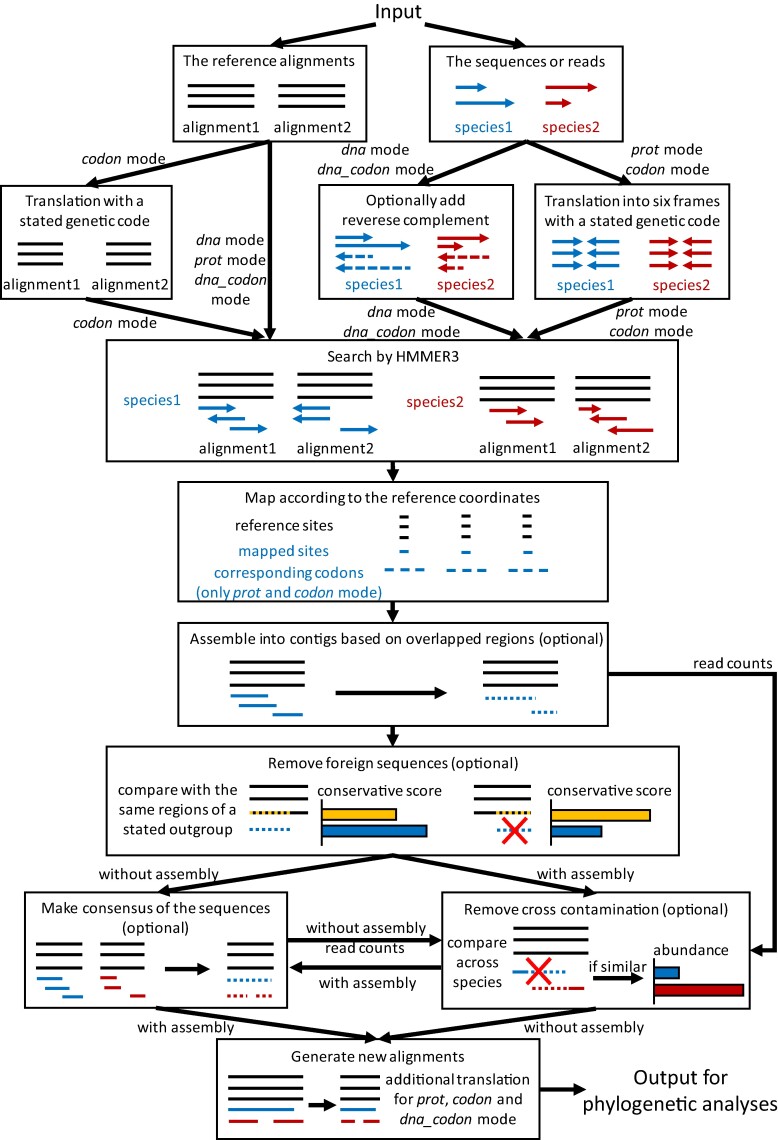
Overview of the strategy and technological processes employed by PhyloAln.

To streamline the intricate and time-consuming process of preparing MSAs using omic data for phylogenetic and other evolutionary analyses, we introduce PhyloAln. It is a command line tool based on Python framework and designed to map not only assembled sequences but also raw reads onto reference MSAs, accommodating various data types (Fig. 1). The workflow begins with the preprocessing of reference MSAs and target sequences or reads, incorporating optional reverse complement and translation based on user-defined modes. Subsequently, the processed sequences and reads undergo a search against the reference MSAs using HMMER3 software (Mistry et al. 2013), and the mapped sequences are aligned to the reference coordinates derived from HMMER3 search results. These mapped sequences undergo optional assembly, foreign and cross-contamination removal, and consensus steps. Finally, the resulting mapped sequences are included in the output DNA and protein MSAs, providing new MSA files suitable for downstream phylogenetic analyses. Further details for each step are outlined in the Materials and Methods section.

### Performance on Simulated Data across Tree of Life with Different Coverages

**Fig. 2.**
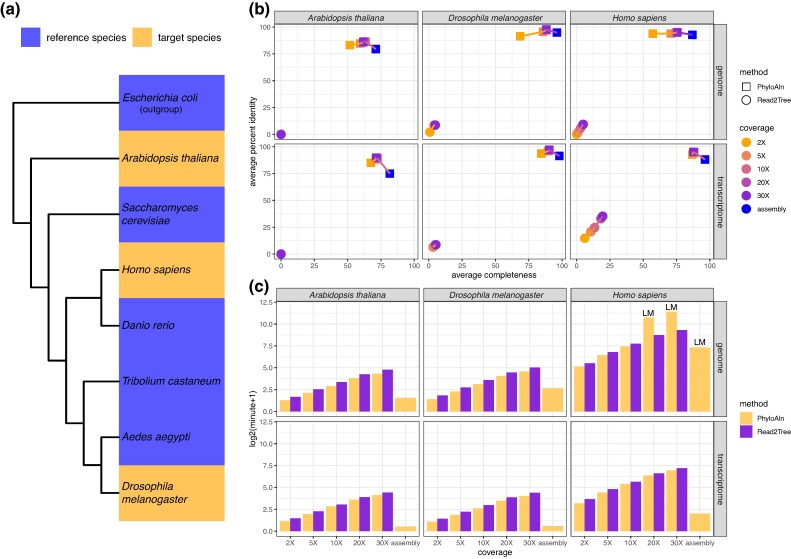
Performance test of PhyloAln and Read2Tree on the simulated data set across the tree of life. a) Design for the data set. Reference species were used to generate the reference MSAs for PhyloAln, and target species were used to provide the sources of reads or assemblies to be mapped onto the reference MSAs. b) Average completeness and percent identity of the MSAs generated by PhyloAln and Read2Tree using simulated Illumina reads or original assemblies of genomes and transcriptomes from three target species with varying coverages. MAFFT MSAs based on *de novo* gene orthology assignment by OrthoFinder were used as standard references. c) Running time of PhyloAln and Read2Tree using simulated Illumina reads or original assemblies of genomes and transcriptomes from three target species with different coverages. LM: using a slower low-memory strategy to prepare the sequences and reads because of the large size of the data.

We conducted a comparative analysis of the MSA performance between PhyloAln and another reference-based tool, Read2Tree (Dylus et al. 2024), utilizing simulated reads encompassing different omic data (genomes and transcriptomes), sequencing technologies (Illumina, PacBio, and Nanopore), coverages (2×, 5×, 10×, 20×, 30×, and complete assembly sequences), and species across the tree of life (*Arabidopsis thaliana*, *Homo sapiens*, and *Drosophila melanogaster*; Fig. 2a). In the case of short Illumina reads, PhyloAln consistently outperformed Read2Tree, yielding more complete and identical MSAs of single-copy orthologous genes (average completeness: 51.61% to 90.44%, average identity: 83.19% to 97.82%, of all genes) in the three target species, with the MAFFT (Katoh and Standley 2013) MSAs based on *de novo* orthology assignment using gene predictions from the assembly sequences as standard references (Fig. 2b). Read2Tree exhibited lower performance (average completeness: 0% to 19.55%, average identity: 0% to 35.25%). PhyloAln demonstrated comparable or even shorter runtime across most scenarios (1.13 to 175.25 min, except for 1717.44 and 2728.22 min for the extremely large genomic data of *H. sapiens* with the coverage of 20× and 30×, respectively, using the slower low-memory strategy) in comparison to Read2Tree (1.70 to 628.94 min) for aligning Illumina short reads (Fig. 2c; supplementary table S2, Supplementary Material online). Moreover, in contrast to Read2Tree, which works only with reads, PhyloAln efficiently mapped assembly sequences directly into MSAs with high completeness (71.12% to 97.99%) and identity (74.99% to 94.88%), requiring minimal time (0.44 to 5.34 min, except 158.52 min for *H. sapiens* genome using the low-memory strategy). For long PacBio and Nanopore reads, PhyloAln demonstrated the capability to generate MSAs with relatively high completeness (75.95% to 96.37%) and moderate identity (45.49% to 84.52%) (supplementary figs. S1 and S2, Supplementary Material online).

### Ability to Remove Foreign and Cross-contamination

**Fig. 3.**
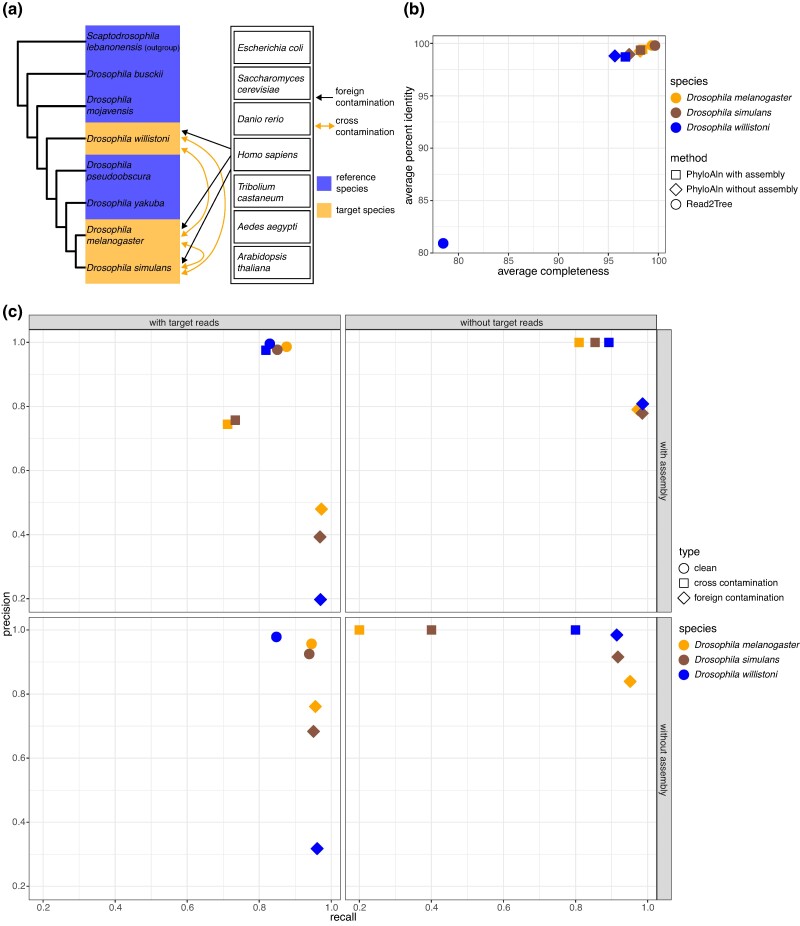
Performance test of PhyloAln and Read2Tree on the simulated data set of contaminated fruit fly transcriptomes. a) Design for the data set. Reference species were used to generate the reference MSAs for PhyloAln, and target species were used to provide the sources of reads or assemblies to be mapped onto the reference MSAs. b) Average completeness and percent identity of the MSAs generated by PhyloAln and Read2Tree using simulated reads of contaminated transcriptomes from three target fruit fly species. MAFFT MSAs based on *de novo* gene orthology assignment by OrthoFinder were used as standard references. c) Precision and recall of PhyloAln to align the reads from the corresponding target species (clean reads) and remove the reads of foreign contamination and cross-contamination with or without the assembly step and the target reads.

We evaluated the capability of PhyloAln to eliminate foreign and cross-contamination, which are common issues in sequencing data (Lusk 2014; Borner and Burmester 2017; Simion et al. 2018) often overlooked by other reference-based tools. This assessment was conducted using a simulated data set of contaminated fruit fly transcriptomes (Fig. 3a). Using the simulated data set, we conducted a comparative analysis of the overall performance on the MSAs generated by PhyloAln and Read2Tree. Regarding the completeness and identity of the MSAs, PhyloAln both with and without the assembly step based on the reference coordinates demonstrated strong performance. The assembly step exhibited slightly superior MSAs (average completeness: 96.70% to 98.42%, average identity: 98.70% to 99.43% with the assembly step vs. average completeness: 95.64% to 98.17%, average identity: 98.81% to 99.26% without the assembly step; Fig. 3b). Read2Tree also exhibited good performance on the aligned sequences of *D. melanogaster* and *Drosophila simulans* (average completeness: 99.34% to 99.65%, average identity: 99.79% to 99.80%). However, its performance was relatively weaker on the sequences of *Drosophila willistoni* (average completeness: 78.47%, average identity: 80.92%).

To assess decontamination efficacy of PhyloAln, we quantified the precision and recall of aligned reads (clean), as well as reads removed during the foreign and cross-contamination removal steps, categorized by their species sources. PhyloAln consistently demonstrated high precision (0.925 to 0.996) and recall (0.829 to 0.945) in aligning clean reads from the target species, both with and without the assembly step (Fig. 3c). In orthologous genes with target reads in the data, PhyloAln successfully removed 95.07% to 97.27% of foreign contamination with a precision of 0.197 to 0.761, irrespective of the assembly step. It also eliminated 71.21% to 81.85% of cross-contamination with a precision of 0.744 to 0.976 when the assembly step was applied. Notably, PhyloAln without the assembly step did not detect any cross-contamination reads, likely due to their coverage by the consensus with a substantial number of clean reads, resulting in minimal impact on the output MSAs (Fig. 3b). In the five orthologous genes where we retained only contamination reads by removing target reads, PhyloAln, with or without the assembly step, successfully removed 91.44% to 98.63% of foreign contamination reads with a precision of 0.779 to 0.984. However, the assembly step detected 80.97% to 89.27% of cross-contamination, compared to 20% to 80% detected without this step.

Upon observing relatively low precision in foreign contamination detection using PhyloAln, with or without the assembly step, in orthologous genes with target reads, we examined the MSAs generated by the consensus specifically for reads detected as foreign contamination but originating from the target species. We observed that these mapped reads in the MSAs exhibited poor alignment quality, mostly with lower percent identity compared to the output MSAs of PhyloAln (32 to 35 out of 41 MSAs with retained target reads; supplementary figs. S3 and S4, Supplementary Material online). This suggests that these poorly aligned reads are primarily attributed to paralogous genes, randomly similar sequences, or hypervariable regions, making limited contributions to a robust phylogeny. The phylogenetic trees reconstructed from these poor alignments, which exhibited incorrect positions for the three target species, further support our inference (supplementary fig. S5, Supplementary Material online).

In addition, we also assessed the influence of outgroup selection on the resulting MSAs, in order to avoid significant performance declines caused by inappropriate outgroup selection in PhyloAln. Substituting the outgroup from *Scaptodrosophila lebanonensis* with other reference species of *Drosophila* resulted in minimal changes to performance of the resulting MSAs and decontamination efficacy (average completeness < 3%, average identity < 0.6%, precision and recall of clean reads < 0.04; supplementary table S3, Supplementary Material online). These findings demonstrate minimal impact of outgroup selection in the reference MSAs within a specific taxonomic group, which indicates the robustness of our method in outgroup selection for removing foreign contamination from species distantly related to the reference species.

### Performance on the Real Data Set of the Ladybird Beetle Transcriptomes

**Fig. 4.**
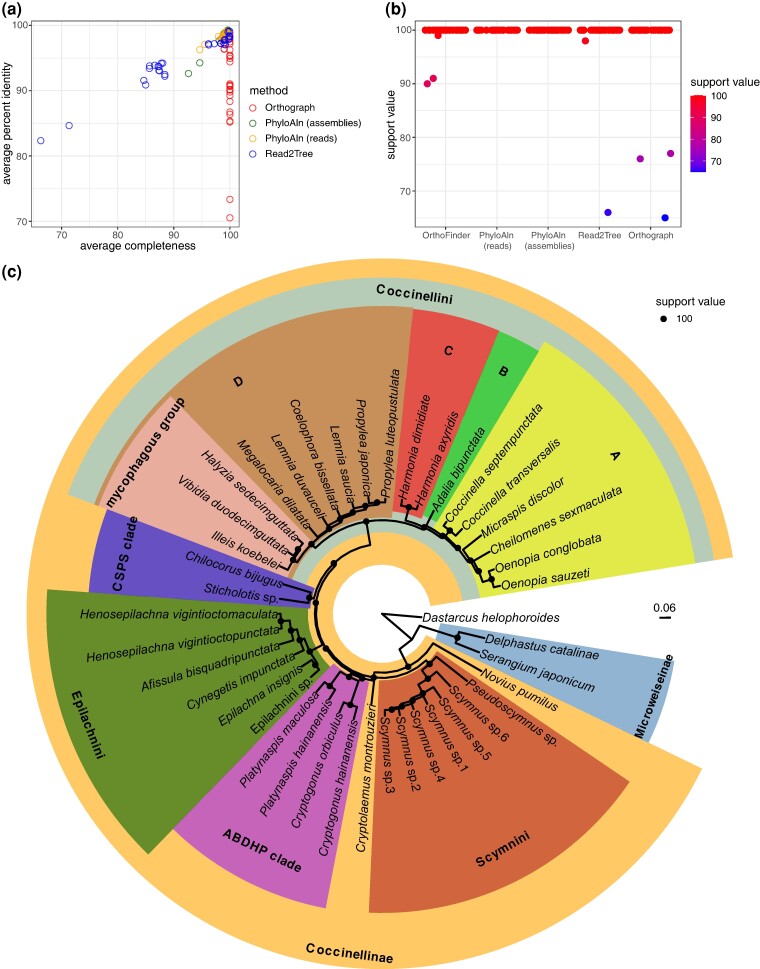
Performance test of PhyloAln, Read2Tree, and Orthograph on the real data set of ladybird beetle transcriptomes. a) Average completeness and percent identity of the MSAs generated by PhyloAln using the reads or assemblies, Read2Tree using the reads, and Orthograph using the assemblies from the 30 target ladybird transcriptomes. MAFFT MSAs based on *de novo* gene predictions and orthology assignment by OrthoFinder were used as standard references. b) The UFBoot support values of all nodes in the phylogeny reconstructed by IQ-TREE based on the MSAs generated by different methods. The MSAs were respectively obtained through *de novo* gene predictions and orthology assignments by OrthoFinder, PhyloAln using the reads or the assemblies, Read2Tree using the reads, and Orthograph using the assemblies. c) The robust phylogenetic tree of the ladybird transcriptomes reconstructed by IQ-TREE based on the MSAs generated by PhyloAln using the reads, with the same topology as those produced through PhyloAln using the assemblies. The reported monophyletic clades in the ladybird beetles (Che et al. 2021; Nattier et al. 2021; Tomaszewska et al. 2021; Li et al. 2021b) include Microweiseinae, Coccinellinae, Coccinellini, Epilachnini, ABDHP Clade, CSPS Clade, Scymnini, mycophagous group, and four clades (A to D) in Coccinellini.

Furthermore, we evaluated the performance of PhyloAln using both reads and assemblies, Read2Tree employing reads, and Orthograph (Petersen et al. 2017) utilizing assemblies on a real data set of ladybird beetle transcriptomes (Li et al. 2021b). PhyloAln effectively mapped reads and assemblies to the reference MSAs, consistently achieving high completeness (94.63% to 99.58% with reads and 92.61% to 99.65% with assemblies) and identity (96.26% to 99.13% with reads and 92.63% to 99.27% with assemblies). In contrast, Read2Tree results exhibited completeness ranging from 66.29% to 99.96% and identity ranging from 82.35% to 99.09%, while Orthograph achieved sequence searches with completeness between 98.95% and 100% and identity between 70.54% and 98.85% (Fig. 4a). The subsequent phylogenetic tree reconstructed from the MSAs revealed that all nodes, in both the PhyloAln-based trees using reads or assemblies, were identical and highly supported, with an ultrafast bootstrap (UFBoot) value of 100 (Fig. 4b). The reported monophyletic clades in ladybird beetles, such as Microweiseinae, Coccinellinae, Coccinellini, Epilachnini, ABDHP Clade, CSPS Clade, Scymnini, mycophagous group, and four clades (A to D) in Coccinellini (Che et al. 2021; Nattier et al. 2021; Tomaszewska et al. 2021; Li et al. 2021b), were all supported in the PhyloAln-based phylogenetic trees (Fig. 4c). Although the topologies of the phylogeny reconstructed based on the MSAs generated by *de novo* gene predictions and orthology assignments by OrthoFinder (Emms and Kelly 2019), PhyloAln using reads or assemblies, Read2Tree using reads, and Orthograph using assemblies differed at a few nodes (supplementary figs. S6 to S10, Supplementary Material online), the unique nodes in the PhyloAln-based trees occurred at those with relatively low support UFBoot values of 65 to 77 in at least one phylogenetic tree using other methods (Fig. 4b; supplementary figs. S6 to S10, Supplementary Material online). Furthermore, the positions of *Megalocaria dilatata* and *Micraspis discolor* in the trees using PhyloAln, conflicting with other methods, are consistent with the reported Coccinellini phylogeny with comprehensive species samples (Nattier et al. 2021; Tomaszewska et al. 2021). All these findings suggest potentially more robust trees using PhyloAln.

### Application on Data of Pepper Plastomes

**Fig. 5.**
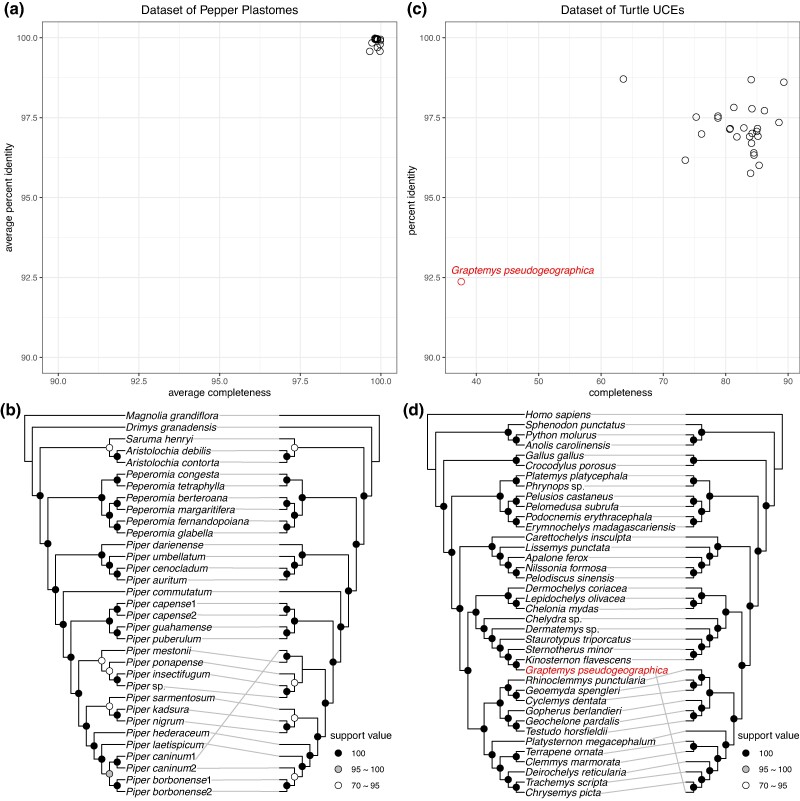
Applications of PhyloAln on the real data sets of pepper plastomes and turtle UCEs. a) Average completeness and percent identity of the MSAs generated by PhyloAln using the reads of 17 target pepper plastomes. b) Comparison of the topologies of the phylogenetic trees reconstructed by IQ-TREE based on the MSAs generated through PhyloAln using the reads (left) and the gene annotations of the plastome assemblies (right). c) Completeness and percent identity of the UCE result MSA generated by PhyloAln using the reads of UCE sequencing data of 27 target turtles. d) Comparison of the topologies of the phylogenetic trees reconstructed by IQ-TREE based on the MSA generated by PhyloAln using the reads (left) and the downloaded concatenated UCE matrix (right). *G. pseudogeographica* is putatively from different sources of reads and aligned sequences in the downloaded concatenated UCE matrix, thus having different positions in the two phylogenetic trees.

To showcase the utility of PhyloAln in handling genes with nonstandard genetic codes, we employed the tool to map reads from pepper plastomes (Simmonds et al. 2021) into reference MSAs and assessed its performance. The resulting MSAs from PhyloAln exhibited remarkably high completeness (99.65% to 99.98%) and identity (99.57% to 99.99%) when compared to MSAs generated from gene predictions of the plastome assemblies as standard references (Fig. 5a). The phylogenetic trees reconstructed from MSAs produced by PhyloAln and gene predictions of the assemblies displayed nearly identical topologies, with the exception of the position of *Piper caninum*1 (Fig. 5b). In the phylogenetic tree generated using PhyloAln, *P. caninum*1 emerged as the sister sample to *P. caninum*2, a relationship that appears more reliable considering the belonging to the same species.

### Application on Data of Turtle UCEs

To evaluate the applicability of PhyloAln on diverse data sets such as UCEs, a category of DNA genetic markers (Faircloth et al. 2012), we utilized the tool to map reads to the reference concatenated UCE matrix of turtles (Crawford et al. 2015). Our tool produced MSA with moderate completeness ranging from 63.58% to 89.32% (except 37.58% for *Graptemys pseudogeographica*) and high identity spanning 95.76% to 98.71% (except 92.37% for *G. pseudogeographica*; Fig. 5c). Phylogenetic trees constructed based on MSA generated by PhyloAln and the published concatenated matrix displayed nearly identical topologies, with the exception of the position of *G. pseudogeographica* (Fig. 5d). However, upon scrutinizing the read data of *G. pseudogeographica* downloaded from National Center for Biotechnology Information (NCBI), we observed that the assembled sequences from these reads (a total of 3,054 sequences with best hits) were primarily closest to *Kinosternon flavescens* (1,418 sequences), followed by *Sternotherus minor* (865 sequences) and *Staurotypus triporcatus* (286 sequences), which were the three closest species to *G. pseudogeographica* in our PhyloAln tree (Figs. 5d; supplementary fig. S11, Supplementary Material online). In contrast, only 12 sequences were closest to the *G. pseudogeographica* sequences in the downloaded supermatrix. Therefore, we concluded that the reads on NCBI and the sequences from the published concatenated matrix originated from different sources, and our PhyloAln tree accurately depicted the correct topology across UCE read sources.

## Discussion

In the current era, an increasing volume of omic data is being sequenced, notably including genomic and transcriptomic data sets rich in gene content, as well as amplicons, mitogenomes, and plastomes, all of which are valuable for phylogenetic analyses. Large-scale initiatives, such as the i5K initiative to sequence 5,000 arthropod genomes (Evans et al. 2013), the Vertebrate Genome Project (Rhie et al. 2021), and the Darwin Tree of Life Project (The Darwin Tree of Life Project Consortium 2022), contribute to the rapid generation of omic data. With the availability of these extensive omic data sets, there is a growing demand for user-friendly pipelines and tools to efficiently process such data for large-scale phylogenetic and evolutionary analyses (Kapli et al. 2020). Reference-based MSA has emerged as a novel and viable strategy, utilizing orthology databases (Altenhoff et al. 2024; Kuznetsov et al. 2023) and manually *de novo* clustered orthology (Li et al. 2003; Emms and Kelly 2019) based on parts of the high-quality genomes to assign genes from omic data. Here, we introduce PhyloAln, a reference-based MSA tool designed for phylogenetic and evolutionary analyses. PhyloAln offers the capability to map various types of sequences or reads into reference MSAs and generate new MSAs. This tool provides a more convenient approach for preparing MSAs and performing downstream phylogenetic or evolutionary analyses, particularly suited for the developing landscape of high-throughput technologies and the increasing volume of omic data, as compared to traditional methods relying on *de novo* assembly, orthology assignments, and MSAs (Kapli et al. 2020).

Based on our tests of completeness and identity, PhyloAln demonstrated effective MSAs of genomic and transcriptomic reads and sequences from different sequencing technologies (Illumina, PacBio, and Nanopore), coverages (2×, 5×, 10×, 20×, 30×, and original assembly sequences), and three model organisms across the tree of life, including an insect, a vertebrate, and a plant. Actually, the performance of *de novo* assembly-based processes is often constrained by low-coverage sequencing data (Liao et al. 2019; Dylus et al. 2024). The reference-based approach of PhyloAln allows it to robustly handle reads with low coverage (at least 2×), enabling phylogenetic analyses with low-coverage omic data and, consequently, offering cost-effective and data-abundant sampling capabilities.

Additionally, Read2Tree, a reference-based tool utilizing expert read aligners, faces challenges in handling target species with divergence larger than hundreds of millions of years from the reference species (Dylus et al. 2024), consequently exhibiting poor performance on the data set of the tree of life. Other sequence search tools like DIAMOND (Buchfink et al. 2021) are mostly based on pairwise alignment of specific query sequences. For candidate dependency of reference MSA-based tools, this search strategy brings an additional problem of how to select the best query sequences to conduct the search and subsequently integrate the search results into the alignments. This also raises our concerns that potential bias of overfitting between the best query sequences and the target sequences would have an impact on the global alignments and downstream analyses. Instead of the read aligners and pairwise alignment-based tools, the current version of PhyloAln uses HMMER3 (Mistry et al. 2013), a profile hidden Markov model (pHMM)–based sequence search tool. The pHMM-based strategy of HMMER3 targets the conservative sites in the global MSAs, which is informative to the phylogenetic and evolutionary analyses. This feature makes it one of the most sensitive search tools, surpassing other search tools such as DIAMOND and MMseqs2 (Steinegger and Söding 2017; Steinegger et al. 2019). These advantages of HMMER3 ensure a stable performance of PhyloAln on conservative sites when mapping target species distantly related to the reference species, spanning families, classes, and even kingdoms, with percent completeness and identity at least 50 percentage points higher than those achieved by Read2Tree.

In view of the prevalent issues of foreign and cross-contamination in omic data (Lusk 2014; Borner and Burmester 2017; Simion et al. 2018), we have devised methods to address these challenges and incorporated them into PhyloAln. Through tests with simulated fruit fly transcriptomes with contamination, PhyloAln demonstrated the ability to remove a minimum of approximately 90% of foreign contamination, regardless of whether the assembly step was included. It also exhibited the capacity to eliminate at least around 70% of cross-contamination with the assembly step, ultimately aligning reads from the target species with high precision ranging from 0.925 to 0.996. Without the assembly step, the ability to detect cross-contamination decreased to 20% to 80%, possibly due to the absence of precise read count information during the assembly step and the potential disturbance of final sequence composition by reads from different contamination sources during the consensus. However, this ability is only essential in unusual conditions where corresponding reads from the target species are absent. In most cases, the substantial number of target reads is likely to offset the impact of relatively few contamination reads in the consensus. Consequently, PhyloAln, with or without the assembly step, can both yield MSAs with high completeness and identity. PhyloAln also identifies poorly aligned sequences or reads as foreign contamination and removes them, contributing to a more robust phylogenetic tree compared to other tools.

Unlike other reference-based tools such as Read2Tree (Dylus et al. 2024) and Orthograph (Petersen et al. 2017), which either do not consider the contamination issues or necessitate additional decontamination before operation, PhyloAln is specifically designed to handle these common contamination scenarios. This advantage is evident in the stable performance of PhyloAln in MSAs compared to Read2Tree, particularly under simulated contamination conditions or real data sets of transcriptomes. Though Read2Tree performed very well on some species in these data sets (e.g. *D. melanogaster* and *D. simulans*), sequences of other species in MSAs generated by Read2Tree exhibited noticeably lower completeness and identity, especially for species less related to the reference species in the phylogeny (e.g. *D. willistoni*). It is plausible that the read aligner dependency of Read2Tree tends to map reads close to the reference species, resulting in low completeness and attraction to contamination from species closer to the reference sequences, leading to low identity. In contrast, PhyloAln has a distinct advantage in managing contamination scenarios, demonstrating consistently high performance in MSAs.

Moreover, the application of PhyloAln to data sets encompassing pepper plastomes and turtle UCEs confirms its broad utility in phylogenetic analyses utilizing diverse omic data from various species groups. Phylogenetic studies commonly leverage not only nuclear protein–coding genes derived from genomes or transcriptomes (Cheon et al. 2020; Kapli et al. 2020) but also DNA sequences such as UCEs (Faircloth et al. 2012), as well as ribosomal genes, mitochondrial sequences, and plastid sequences (Patwardhan et al. 2014). Researchers may encounter a spectrum of data types, including sequenced reads, existing assemblies lacking high-quality gene predictions, predicted gene sequences, and even translated protein sequences. The ability to adeptly process diverse sequence types and accommodate different genetic codes represents an additional advantage of PhyloAln, making it well suited for a wide array of phylogenetic and evolutionary analyses.

However, it should be noted that PhyloAln is designed as an exclusively reference-based tool, consequently disregarding regions present in the target species but absent or inconsistent in the reference MSAs. Therefore, it will consistently miss this information, rendering it unsuitable as a substitute for *de novo* assembly-based methods in cases requiring different standards with those for phylogenetic analyses. In comparison with the most user-friendly workflows for preparing reference MSAs and conducting downstream phylogenetic analyses, we also prioritize the flexibility of use in PhyloAln. This allows the tool to accept previously prepared reference MSAs and produce resulting MSAs for user-customized downstream analyses, but basic knowledge of data acquisition, outgroup selection, MSA execution, and downstream phylogenetic or evolutionary analyses is typically required for using PhyloAln. In addition, we have adopted a tentative approach that does not heavily focus on optimizing the runtime and memory usage of PhyloAln. This makes it susceptible to the influence of the number of reference MSAs and the sizes of the target sequences/reads. Consequently, this may restrict its ease of use with extremely large or complex data sets in phylogenetic analyses. To address this limitation, PhyloAln could be further developed by optimizing parallel and storage operations and incorporating optional accessories programmed in languages faster than Python for executing time-consuming steps. Faster sequence search tools (e.g. DIAMOND [Buchfink et al. 2021], MMseqs2 [Steinegger and Söding 2017], and HH-suite [Steinegger et al. 2019]) could also be a candidate to be integrated into PhyloAln as an option and evaluated against HMMER3 to devise a recommended selection strategy in the future.

Overall, our reference-based MSA tool, PhyloAln, stands out by offering a stable and direct mapping capability for sequences or reads from diverse omic data to reference MSAs. Importantly, PhyloAln is designed to ideally remove common contaminations found in sequencing data. It is able to facilitate the streamlined preparation of MSAs for large-scale phylogenetic and evolutionary analyses in the current omic era.

## Materials and Methods

### Technological Processes of PhyloAln

PhyloAln requires one or more reference MSAs in FASTA format and one or more read/sequence files in FASTQ/FASTA format as input. The mapping of sequences into the MSAs relies primarily on a search tool utilizing pHMMs, HMMER3 v3.1b2 (Mistry et al. 2013), with preparation and downstream processes implemented using Python. To accommodate diverse data types, we have designed four search modes, namely, “dna”, “prot”, “codon”, and “dna_codon”. The “dna” mode is tailored for direct HMMER3 search, suitable for nucleotide-to-nucleotide or protein-to-protein alignments, with an optional consideration for the reverse complement of sequences, particularly applicable in nucleotide-to-nucleotide alignments. The “prot” mode and “codon” mode necessitate protein MSAs or codon MSAs as references, respectively. In these modes, protein MSAs directly provided or translated from codon MSAs are employed to search six translation frames of the sequences using HMMER3. The “dna_codon” mode shares similarities with the “dna” mode in the HMMER3 search but additionally generates protein MSAs by compulsively translating the output nucleotide MSAs. This mode is suitable for codon-to-nucleotide alignment, for instance, when mapping long reads with a high ratio of insertions and deletions (Goodwin et al. 2016) causing translation frame shifts into the MSAs. All translations can be executed using standard or nonstandard genetic codes. Moreover, both the reference MSAs and sequences can be optionally fragmented into smaller segments with a specified sliding length and step. This feature is designed for handling long MSAs with time-consuming HMMER3 searches and genomic sequences with discontinuous coding regions, respectively. The HMMER3 search, combined with these preparations, facilitates the MSA of nuclear protein–coding genes, as well as protein-coding sequences with nonstandard genetic codes, non-protein–coding sequences, translated amino acid sequences, and various types of omic data.

Following the HMMER3 search, the matched sequences for each MSA are subsequently extracted individually. Detailed information from the HMMER3 output files is then utilized to map the matched regions, including gaps, of the extracted sequences into corresponding site coordinates within the reference MSAs. In the “prot” mode and “codon” mode, the codons are associated with their respective amino acid sites. Optionally, these matched regions can be assembled into contigs based on overlapping coordinates, with specified overlapped length and percent identity thresholds.

To lessen the impact of foreign contamination and poorly aligned regions, we have developed a conservative scoring method to assess the degree of matching for each target sequence in comparison to a specified outgroup in the reference MSAs. Initially, the frequencies of each specific base at each site within the ingroup reference MSAs (excluding the specified outgroup) are computed and stored for subsequent calculation of conservative score. Next, the conservative scores of each target sequence and the outgroup sequence within the corresponding regions are calculated and compared. These conservative scores are the summation of the aforementioned frequencies of bases at the respective sites in the target sequence or the outgroup sequence. The conservative score of the outgroup can be adjusted by multiplying a weight coefficient, which is default as 0.9. If the conservative score of the target sequence is lower than the adjusted conservative score of the outgroup, the sequence is identified as foreign contamination and subsequently removed.

Multiple sources/species with their respective sequence files can be inputted through a configuration file. The optional detection of cross-contamination across the sources within the same configuration file is facilitated through sequence comparisons between any two sources, following processes similar to the methodology outlined in the CroCo software (Simion et al. 2018). If the overlapped regions between two sequences surpass the specified length and percent identity, the two sequences are flagged as potential cross-contamination, and their abundance is scrutinized. The counts of reads constituting the two sequences are then normalized and compared. Given that reference MSAs always do not encompass the entire gene sets and standard transcript per kilobase per million mapped reads (TPM) and reads per kilobase per million mapped reads (RPKM) values cannot be directly calculated, we utilize an estimated value of RPKM to represent abundance. The estimated RPKM is computed by dividing the read counts by the sequence length in the reference coordinates and the total read count in the read files. If the fold of the estimated RPKM exceeds the specified threshold, the sequence with lower abundance is identified as cross-contamination from the source of the sequence with higher abundance and is subsequently removed.

Consensus of sites at the same reference coordinates and from the same sources can be optionally conducted before outputting the resulting MSAs. Specifically, if the assembly step is disabled, cross-contamination will be detected and removed using the aforementioned method after the consensus of sequences. Ultimately, the aligned target sequences, with or without aligned reference sequences, are output to a new MSA FASTA file. In the “prot”, “codon”, and “dna_codon” modes, translated protein MSAs are also generated. All these steps are executed in parallel for computational efficiency.

### The Simulated Data Set

To assess capability of PhyloAln in mapping sequences/reads into reference MSAs across diverse species spanning the tree of life and its performance on contaminated data, we constructed a simulated data set. This data set comprises genomes from one bacterium (*Escherichia coli* [Hayashi et al. 2001]), one plant (*A. thaliana* [Cheng et al. 2017]), one fungus (*Saccharomyces cerevisiae* [Engel et al. 2022]), two vertebrates (*Danio rerio* [Howe et al. 2013] and *H. sapiens* [Collins et al. 2004]), eight fruit flies (*S. lebanonensis* [Flynn et al. 2020], *D. melanogaster* [Hoskins et al. 2015], *D. simulans* [Wang et al. 2023], *D. willistoni* [Ranz et al. 2023], *Drosophila mojavensis* [Kim et al. 2021], *Drosophila yakuba* [Huang et al. 2022], *Drosophila busckii* [Renschler et al. 2019], and *Drosophila pseudoobscura* [Barata et al. 2023]), and two other insects (*Tribolium castaneum* [Herndon et al. 2020] and *Aedes aegypti* [Matthews et al. 2018]), sourced from the NCBI RefSeq database (Haft et al. 2024) (supplementary table S1, Supplementary Material online). The phylogeny of these species was obtained from the TimeTree database (Kumar et al. 2022). Coding sequences, protein sequences, and transcripts were extracted based on general feature format annotation files. For constructing reference MSAs, *de novo* orthology assignment was performed using OrthoFinder v2.5.4 (Emms and Kelly 2019) on the longest transcript of the coding sequence for each gene in the aforementioned species. Subsequently, 46 single-copy genes among all the species were aligned using the L-INS-i mode of MAFFT v7.480 based on protein sequences (Katoh and Standley 2013). These MSAs were then back-translated to codon MSAs and trimmed using trimAl v1.4 (Capella-Gutierrez et al. 2009) with the “automated1” option to eliminate poorly aligned regions. These trimmed MSAs served as the standard MSAs for evaluating the performance of PhyloAln.

The data set of the tree of life comprises *E. coli*, *S. cerevisiae*, *D. rerio*, *T. castaneum*, and *A. aegypti* as reference species to generate the reference MSAs for PhyloAln, with *A. thaliana*, *H. sapiens*, and *D. melanogaster* selected as target species to provide the sources of the reads or sequences to be mapped into the reference MSAs. The five reference species were extracted from the 46 MSAs, and sites with all gaps were removed to generate the reference MSA files for this data set. The three target species were chosen to represent varying degrees of taxonomic relatedness to the closest reference species, spanning kingdoms (*A. thaliana*), classes (*H. sapiens*) and families (*D. melanogaster*). With the genome or transcript sequences of the target species as templates, simulated Illumina reads, with a length of 100 bp, were generated using ART v2.5.8 (Huang et al. 2012), while Nanopore and PacBio reads were simulated by ReadSim v1.6 (https://sourceforge.net/projects/readsim/) (Lee et al. 2014) with default average length. The coverage levels for genome and transcript sequences of the target species were set at 2×, 5×, 10×, 20×, and 30×, respectively.

For the contaminated data set, transcripts from fruit fly species were mainly utilized, with *S. lebanonensis*, *D. mojavensis*, *D. yakuba*, *D. busckii*, and *D. pseudoobscura* as reference species and *D. melanogaster*, *D. simulans*, and *D. willistoni* as target species. Additionally, *E. coli*, *A. thaliana*, *S. cerevisiae*, *D. rerio*, *H. sapiens*, *T. castaneum*, and *A. aegypti* were included as foreign contamination sources. The reference MSA files for the five reference species were prepared as described above. Illumina reads of the transcripts from target and foreign species were simulated by ART with a coverage of 10×, labeled with the source species for downstream testing. For each target species, reads were randomly selected 15,000,000 to 20,000,000 times. These reads were combined with 10,000 to 500,000 random reads from each foreign species and two other target species to introduce simulated foreign and cross-contamination, respectively. To evaluate performance of the tools on sequences contaminated without being covered by large amounts of real target reads in the sequence consensus, 5 of the 46 single-copy genes in each target species were respectively selected to remove reads from the target regions while retaining the contamination reads.

### The Real Data Set of Ladybird Beetle Transcriptomes

We selected genomes of 11 ladybird beetle (Coccinellidae) species (*Adalia bipunctata* [Wellcome Sanger Institute Tree of Life Programme et al. 2022], *Coccinella septempunctata* [Crowley et al. 2021], *Cryptolaemus montrouzieri* [Li et al. 2021a], *Cynegetis impunctata* [our unpublished data, NCBI accession: GCA_030704885.1], *Halyzia sedecimguttata* [Crowley et al. 2023], *Harmonia axyridis* [Boyes et al. 2021], *Henosepilachna vigintioctomaculata* [Zhu et al. 2023], *Henosepilachna vigintioctopunctata* [our unpublished data, NCBI accession: GCA_030704895.1], *M. discolor* [our unpublished data, NCBI accession: GCA_030674115.1], *Novius pumilus* [Tang et al. 2022], and *Propylea japonica* [Zhang et al. 2020]) and one Coccinelloidea outgroup *Dastarcus helophoroides* (Zhang et al. 2023), as reference species (supplementary table S1, Supplementary Material online). The genomes were downloaded from the NCBI Genome database. Gene predictions for *C. septempunctata* and *H. axyridis* were obtained from the NCBI RefSeq database, while those for the other ten genomes were generated using a modified FunAnnotate v1.8.1 pipeline (https://github.com/nextgenusfs/funannotate), following the methodology outlined by Tang et al. (2022).

The Illumina read files for 30 target ladybird beetle transcriptomes were obtained from the supplementary NCBI SRA accessions provided by Li et al. (2021b) (supplementary table S1, Supplementary Material online). The data were independently *de novo* assembled using Trinity v2.8.5 (Grabherr et al. 2011). CroCo v1.1 (Simion et al. 2018) was employed with default parameters to identify and eliminate cross-contamination sequences within the same batch. Additionally, a customized nucleotide sequence database for Coccinelloidea was created, encompassing all ladybird genome sequences used in this study, alongside previously published versions (Ando et al. 2018; Gautier et al. 2018; Chen et al. 2021), and the sequences of species under Coccinellidae, Endomychidae, Corylophidae, Latridiidae, Alexiidae, Cerylonidae, Discolomatidae, and Bothrideridae in the NCBI NT database. Subsequently, transcriptomic assemblies were queried against both this customized database and the NCBI NT database using BLAST v2.8.1+ (Camacho et al. 2009). Transcripts with a percent identity < 90% compared to those within our customized database and a percent identity ≥ 98% compared to those in the NCBI NT database were deemed foreign and consequently removed from the assemblies. Nonredundant coding gene sets for each species were constructed using EvidentialGene v2018.06.18 (Gilbert 2013).

Orthology assignment, MSA, and trimming were conducted across all 42 species as outlined for the simulated data set. The 30 target species were then excluded from the MSAs of a total of 1,290 single-copy genes across 12 genomes. The resulting new MSAs, devoid of all-gap sites, were utilized as reference MSAs.

### The Real Data Set of Pepper Plastomes

For plant plastomes, we utilized the data set from Simmonds et al. (2021) to evaluate PhyloAln. The raw Illumina reads of 17 plastomes, along with the gene predictions and assemblies of all 36 plastomes (inclusive of the 17 target plastomes), were acquired from NCBI, based on the supplementary accessions or directly from the supplement materials (supplementary table S1, Supplementary Material online). Protein sequences of 68 single-copy protein-coding genes across all 36 plastomes were aligned using the L-INS-i mode of MAFFT and subsequently back-translated to codon MSAs using the genetic code of 11 (the plant plastid code). The reference MSAs, excluding the 17 target plastomes, were prepared as described earlier.

### The Real Data Set of Turtle UCEs

The turtle UCE data set from Crawford et al. (2015) was employed to assess PhyloAln. The concatenated UCE matrix, encompassing a total of 38 species, was obtained from the Supplementary Material. Raw Illumina reads for 27 target species were retrieved from the NCBI SRA database using the provided supplementary accessions (supplementary table S1, Supplementary Material online). Reference MSAs, excluding the target sequences, were prepared as previously described.

### Run With PhyloAln, Read2Tree, and Orthograph

In the simulated data set spanning the tree of life, reads with varying coverages of genome and transcript sequences from *A. thaliana*, *H. sapiens*, and *D. melanogaster* were aligned to the reference MSAs using PhyloAln and Read2Tree v0.1.5 (Dylus et al. 2024), respectively. *E. coli* was designated as the outgroup in PhyloAln. Due to the extensive memory requirements for the preparation of a large number of sequences in deep-depth read files on our computer server, especially for *H. sapiens*, a slower but less-memory-intensive preparation method, without storage of the sequences in the Python dictionary, was developed in PhyloAln and employed for these tests involving large-depth reads. For Illumina reads, the “codon” mode was used in PhyloAln, while Read2Tree was run with default parameters. For PacBio and Nanopore long reads, the “dna_codon” mode of PhyloAln was used to generate the MSAs. When mapping the long reads of the genomes, the reads were split into fragments of 200 bp with a sliding step of 100 bp. Read2Tree for long reads also split the reads into fragments of 200 bp with ngmlr parameters for PacBio and Nanopore reads, respectively. Additionally, “codon” mode without assembly of PhyloAln was used to align the complete sequences of the genome and transcript sequences to the reference MSAs. Specifically, the genome sequences were initially split into fragments of 200 bp with a sliding step of 100 bp to lessen the impact of intron regions.

Contaminated transcriptomic reads from *D. melanogaster*, *D. simulans*, and *D. willistoni* were aligned to the reference MSAs of fruit flies using both PhyloAln and Read2Tree. Specifically, for PhyloAln, “codon” mode with and without assembly was employed, using *S. lebanonensis* as the outgroup. To assess the influence of different defined outgroups, other four reference species (*D. mojavensis*, *D. yakuba*, *D. busckii*, and *D. pseudoobscura*) were set as outgroup, and PhyloAln was executed with the same parameters for each species.

In the real data set of ladybird beetle transcriptomes, PhyloAln, Read2Tree, and Orthograph v0.7.2 (Petersen et al. 2017) were employed. Due to the large number of reference MSAs, PhyloAln was executed using “codon” mode without assembly. Reads and assemblies after decontamination were mapped into the reference MSAs, respectively, with *D. helophoroides* set as the outgroup in PhyloAln. Read2Tree and Orthograph were run with default parameters, utilizing reads and decontaminated assemblies, respectively.

In addition, the performance of PhyloAln was evaluated using data sets of pepper plastomes and turtle UCEs. For pepper plastomes, assembly-free “codon” mode with the genetic code of 11 was employed. Due to the presence of closely related and even the same species in the target list, making cross-contamination detection challenging (Simion et al. 2018), the step of cross-contamination removal was disabled. *Magnolia grandiflora* was selected as the outgroup following the specifications of the pepper plastome data set (Simmonds et al. 2021). For the concatenated UCE matrix, the reference MSA was initially split into short MSAs of 1,000 bp, and reads were then mapped into these short MSAs using the “dna” mode without assembly. Subsequently, all new short MSAs were concatenated into the final matrix, with all processes executed within PhyloAln. *H. sapiens* was set as the outgroup based on the phylogenetic tree presented in the turtle UCE article (Crawford et al. 2015).

PhyloAln directly utilized the prepared reference MSAs in the data set, while the unaligned sequences of each orthologous gene were provided to Read2Tree and Orthograph. All runs of PhyloAln, Read2Tree, and Orthograph used 20 threads and a maximum memory allocation of 200 GB, and their execution time was recorded using the “time” command in the CentOS Linux 7 system.

### Performance Test of PhyloAln, Read2Tree, and Orthograph

We calculated the completeness and percent identity of each sequence generated by PhyloAln, Read2Tree, and Orthograph in the MSAs based on the reference coordinates. Only the sites with bases or intermediate gaps in the standard reference sequences in the MAFFT MSAs and the generated sequences were considered valid regions, while the unknown or unmapped sites or those with start or end gaps were ignored. Completeness was defined as the percentage of overlapped valid regions in the valid regions of the standard reference sequences. Percent identity was defined as the percentage of the same site in both sequences among the overlapped valid regions. For PhyloAln, the generated sequences in the resulting MSAs were compared with the corresponding sequences in the *de novo* assembled and MAFFT-aligned standard MSAs before removal of the target sequences. Read2Tree generated the reference MSAs itself in the pipeline, and we thus mapped the corresponding assembled sequences into these reference MSAs using the “--add” and “--keeplength” option and L-INS-i mode of MAFFT to obtain the standard MSAs. The sequences in the Read2Tree result MSAs were then compared with the corresponding sequences in the standard MSAs. For Orthograph, since only the unaligned target sequences were obtained, we mapped the result sequences into the reference MSAs of PhyloAln using the “--add” and “--keeplength” option and L-INS-i mode of MAFFT. Subsequently, we evaluated these newly generated MSAs as same as the assessments of PhyloAln MSAs above. Specifically, in the data set of ladybird transcriptomes, only 121 single-copy orthologous genes across all the 42 species, based on orthology assignments by OrthoFinder, were used for the test of completeness and percent identity. This was because these three tools only generated single-copy MSAs in those 30 target species, making it challenging to compare with genes having multiple-copy sequences from the transcriptome assemblies in OrthoFinder results.

To assess the ability of PhyloAln to remove contamination, we utilized the species label of the reads and quantified the reads from each species composing the retained or removed sequences. We calculated the values of true positive (TP), true negative (TN), false positive (FP), false negative (FN), precision [= TP/(TP + FP)], and recall [= TP/(TP + FN)] for three aspects (clean, foreign contamination, and cross-contamination) in each target species. The theoretical and observed positive/negative values were defined as follows:

#### Clean Aspect

The counts of reads from the target species were considered as theoretical positive values.

The counts of reads from other species were considered as theoretical negative values.

Reads retained in the final MSAs were considered as observed positive values.

Reads removed were considered as observed negative values.

#### Foreign Contamination Aspect

The counts of reads from the foreign species were considered as theoretical positive values.

The counts of reads from the three target species were considered as theoretical negative values.

Reads removed in this step were considered as observed positive values.

Reads retained were considered as observed negative values.

#### Cross-contamination Aspect

The counts of reads from the other two target species were considered as theoretical positive values.

The counts of reads from the source species were considered as theoretical negative values.

Reads removed in this step were considered as observed positive values.

Reads retained were considered as observed negative values.

Due to relatively high FP values in foreign contamination detection (putative foreign contamination from the target species), we additionally examined these putative contamination sequences. MSAs containing only these putative contamination sequences were output, and completeness and percent identity were calculated as described above. Protein and codon MSAs of PhyloAln results and the putative contaminations were concatenated into a supermatrix and imported into the “MFP + MERGE” mode of IQ-TREE v2.1.4-beta (Minh et al. 2020) with 1,000 UFBoot replicates, respectively.

In the three real data sets, final phylogenetic trees based on the results generated by PhyloAln, Read2Tree, and Orthograph were reconstructed using IQ-TREE with 1,000 UFBoot replicates. For Orthograph, the result sequences were initially aligned using the L-INS-i mode of MAFFT. The resulting MSAs from the three tools were concatenated into a supermatrix and imported into the “MFP + MERGE” mode of IQ-TREE. Protein MSAs were used in the ladybird transcriptome data set, while codon MSAs were employed in the pepper plastome data set. In the UCE matrix data set without partition information, the concatenated MSA output by PhyloAln was utilized for phylogenetic analysis by IQ-TREE with 1,000 UFBoot replicates and default parameters. Standard trees were constructed based on concatenated protein supermatrix comprising 1,290 single-copy genes from OrthoFinder results in the ladybird transcriptome data set, concatenated codon supermatrix consisting of 68 plastid protein–coding genes from pepper plastome assemblies, and the downloaded UCE matrix. All the trees were rooted with the corresponding outgroup mentioned in PhyloAln processes using the Python ETE 3 Toolkit (Huerta-Cepas et al. 2016) and visualized with the R package ggtree (Yu et al. 2017).

### Check of the Source of *G. pseudogeographica* Reads in the Turtle UCE Data Set

Due to the different positions of *G. pseudogeographica* between the phylogenetic trees obtained from PhyloAln results using read files and the downloaded sequence matrix, along with the relatively low completeness and percent identity of the *G. pseudogeographica* sequence in the test, we investigated the source of its reads. We employed IDBA-UD v1.1.3 (Peng et al. 2012) to assemble the reads, and the resulting assembly was used to search for sequences of all the species in the downloaded sequence matrix using BLAST v2.8.1+ (Camacho et al. 2009) with an *E*-value threshold of 1*e*−5. The species with the best hits for each sequence in the assembly were then counted.

## Supplementary Material

msae150_Supplementary_Data

## Data Availability

References and all accessions of the genomes and reads used are listed in supplementary table S1 of supplementary file S2, Supplementary Material online. The source code for PhyloAln is available under an MIT open-source license at https://github.com/huangyh45/PhyloAln. The detailed data, result files, scripts, commands, and parameters used in the analyses of this research can be found at https://github.com/huangyh45/PhyloAln_manu.
